# Screening for unilateral aldosteronism should be combined with the maximum systolic blood pressure, history of stroke and typical nodules

**DOI:** 10.1097/MD.0000000000031313

**Published:** 2022-10-28

**Authors:** Yumin Meng, Kequan Chen, Aixin Xie, Yueying Liu, Jiangnan Huang

**Affiliations:** a Department of Cardiology, the First Affiliated Hospital of Guangxi Medical University, Nanning, China.

**Keywords:** adrenal venous sampling, computed tomography, history of stroke, maximum systolic blood pressure, unilateral primary aldosteronism

## Abstract

To determine factors associated with lateralization in primary aldosteronism (PA). The clinical data for PA patients hospitalized at the First Affiliated Hospital of Guangxi Medical University from October 2016 to March 2021 were included in this study. They were classified according to results derived from computed tomography (CT): bilaterally normal nodules (no typical nodules were found in either adrenal glands, only changes in unilateral adrenal hyperplasia thickening or bilateral adrenal hyperplasia thickening), unilateral nodules (typical nodule appears in unilateral adrenal gland, and there are no abnormalities in the contralateral adrenal gland or only thickening of unilateral adrenal hyperplasia) and bilateral nodules (typical nodule like changes in bilateral adrenal glands). Multivariate logistic regression and receiver operating characteristic (ROC) were used to analyze the factors associated with lateralization of PA and consistencies between adrenal CT images and adrenal venous sampling (AVS) results. A total of 269 patients with PA were recruited, with an average age of 46 years and 112 cases had typical nodules. Results from CT scans revealed that there were 49 bilateral normal cases, 177 cases were unilateral abnormal and 43 cases were bilateral abnormal. In all of the PA patients, multifactorial logistic regression analysis showed that the maximum systolic blood pressure (OR = 1.03, *P* < .001), history of stroke (OR = 2.61, *P* = .028), and typical nodules (OR = 1.9, *P* = .017) were all relevant factors in unilateral primary aldosteronism (UPA). In the unilateral nodule group, multivariate logistic regression analysis suggested that maximum systolic blood pressure (OR = 1.03, *P* < .001) and typical nodules (OR = 2.37, *P* = .008) were the related factors for UPA. However, the consistency between adrenal CT and AVS was only 40.68%, while maximum systolic blood pressure (OR = 1.02, *P* < .001) and plasma aldosterone renin ratio (OR = 1.001, *P* = .027) were the relevant consistent factors between AVS and CT results. Maximum systolic blood pressure, typical nodules, and history of stroke are important factors to consider when screening for UPA. It is recommended to combine medical history and imaging findings when looking at different subgroups before a clinical decision is made. Patients with PA in the absence of lesions or bilateral lesions on CT should be diagnosed by AVS as far as possible.

## 1. Introduction

Primary aldosteronism (PA) is a disorder of the endocrine system caused by increased secretion of aldosterone in the globular region of the adrenal cortex.^[1]^ It is mainly characterized as the spontaneous production of excess aldosterone, resulting in sodium retention, plasma renin inhibition, increased K + excretion, and elevated blood pressure.^[2]^ Aldosteronoma is a kind of mineralocorticoid synthesized by the glomerular zone (ZG) of the adrenal cortex, which regulates the electrolyte balance through the reabsorption of water and sodium by the kidney. Its biosynthesis is controlled by two main factors: angiotensin II (Ang II) and extracellular potassium concentration (K+).^[3,4]^ Unilateral primary aldosteronism (UPA) is mainly caused by aldosteronogenic adenomas, due to the mutations in potassium and calcium channels or ion pumps. This may lead to membrane depolarization, an elevation of intracellular calcium, disruption of intracellular ion homeostasis and increased adrenal cortical hormone secretion and CYP11B2 (aldosterone synthase) transcription.^[5–7]^ The incidence of PA in patients with hypertension is greater than 10% and approximately 65.6% of secondary hypertension is caused by PA, which is the most common cause of secondary hypertension.^[8–10]^ According to the 2020 European Society of Hypertension expert consensus on primary aldosteronism, the risk of cardiac, renal and cerebrovascular complications and major cardiovascular events in PA patients without good treatment, is significantly higher than that those with primary hypertension, metabolic abnormalities or bone disease.^[11]^

In order to prevent aldosterone mediated end organ injury, it is necessary to improve the ability to diagnose subtypes of PA. This will help to optimize treatment methods and assist clinical management. The two subtypes of PA mainly include unilateral primary aldosteronism (UPA) caused by aldosteronoma and bilateral primary aldosteronism (BPA), which account for more than 95% of all PA cases.The corresponding clinical treatment is unilateral adrenalectomy or surgery combined with pharmacological and antihypertensive drug therapy using salt corticosteroid receptor antagonists.^[2,12,13]^ Adrenal vein sampling (AVS) was first performed in the 1960s,^[14]^ and has been further improved in recent years with the development of imaging. AVS can be performed by cannulating a femoral or upper limb vein and placing the catheter port in bilateral adrenal veins to collect blood and measure aldosterone levels in both adrenal veins, which is used to determine whether aldosterone hypersecretion is unilateral or bilateral. At present, it is considered as the gold standard for the diagnosis of PA subtypes,^[15]^ and its accuracy is reported to be as high as 79% to 100%.^[16]^ However, AVS has only been performed in a limited number of centers and adrenal CT is often used as an alternative method for functional diagnosis.^[17]^ Previous studies have shown that there are numerous differences in results between adrenal CT and AVS and clinical decisions based only on CT imaging may lead to an increased risk of misdiagnoses.^[18]^ However, AVS is invasive, expensive, and requires a high level of technology. Therefore, to perform an accurate and targeted diagnosis and classification of the disease has become the focus of clinical research in this area. This study retrospectively analyzed the clinical data from patients with PA diagnosed at the First Affiliated Hospital of Guangxi Medical University, in order to explore the relationship between AVS lateralization and consistency between CT and AVS results to assist clinicians in the early screening of UPA, which is conducive to the diagnosis and treatment of functional typing of PA patients.

## 2. Method

### 2.1. Patients

In this study, patients with PA treated with AVS were selected as research subjects, and patients hospitalized in the First Affiliated Hospital of Guangxi Medical University from October 2016 to March 2021 were retrospectively included. This study was reviewed by the Medical Ethics Review Committee of the First Affiliated Hospital of Guangxi Medical University, all patients signed written informed consent. The diagnosis of PA was based on criteria from expert consensus on PA in 2020^[17,19]^: using plasma aldosterone-to-renin ratio (ARR) > 20 (ng/dL)/(ng/mL^/^h) (1 ng/dL = 27.7 pmol/L, 1 ng/mL/h = 12.8 pmol/L^/^min), and the diagnosis of PA was confirmed by the Saline infusion test (SIT),or Captopril Challenge test (CCT). SIT: patients started at 8:00 am. and kept sitting for at least 1 hour before and during intravenous infusion of 2 L of 0.9% normal saline within 4 hours. The blood samples for detection of renin, aldosterone, cortisol and plasma potassium were taken at zero time and 4 hours later. During the test, the patient remained seated and monitored blood pressure and heart rate every hour. CCT: Patients received 25 to 50 mg of captopril orally from 8:00 to 9:00 am after sitting or standing for at least 1 hour. Blood samples were withdrawn for PRC and PAC measurement at baseline and 2 hours after provocation, with patients remaining seated during this time. Both SIT and CCT were used to confirm the diagnosis of PA. The 2016 TES guidelines recommend PAC > 10 ng/dL after SIT in the recumbent position as more likely to be PA, <5 ng/dL to exclude PA; PAC > 11 ng/dL after CCT with renin at suppressed levels or ARR > 20 (ng/dL)/[μg/(L/h)] to confirm the diagnosis of PA. For patients diagnosed with PA, AVS and sequential cannulation were performed, and successful catheter insertion was defined as a selectivity index (adrenal to peripheral venous cortisol ratio) ≥ 2. If they showed lateralization of aldosterone secretion during AVS, they were further classified into the UPA group, which showed a lateralization index (aldosterone to cortisol ratio between dominant and nondominant adrenal glands) ≥ 2. Meanwhile, patients with a lateralization index < 2 were classified into the BPA group.^[17]^ AVS was performed according to the consensus from adrenal vein sampling experts,^[2]^ and during hospitalization, adrenal CT enhancement scanning was improved. Patients with unsuccessful AVS blood collection, inability to obtain CT imaging results, or imaging considerations, or pathology confirming a non-adrenocortical origin of the tumor were excluded.

According to the CT findings, patients were divided into: bilateral normal (no typical nodules in both adrenal glands, only changes in unilateral adrenal hyperplasia and thickening or bilateral adrenal hyperplasia and thickening), unilateral nodule (typical nodules in one adrenal gland and no abnormality in the contralateral adrenal gland or only unilateral adrenal hyperplasia and thickening) and bilateral nodule (typical nodule-like changes in both adrenal glands) at the same time, according to AVS results. The results from AVS, were divided into two groups: the group with a secretory dominant side and the group with no secretory dominant side.

### 2.2. Data collection

Detailed data of all patients were collected, including sex, age, body mass index (BMI) ≥ 24, maximum systolic blood pressure, maximum diastolic blood pressure, duration of hypertension, family history of hypertension, dyslipidemia, hyperuricemia, history of stroke, lowest serum potassium, glomerular filtration rate (GFR), endogenous creatinine clearance rate (Ccr), aldosterone at screening, plasma renin activity (PRA) at screening, ARR at screening, and left ventricular mass index (LVMI). Thin slice CT enhanced scanning of the adrenal gland used in this study was interpreted by two professional imaging doctors and a typical adenoma was defined as a unilateral nodule of at least 8 mm in diameter with a smooth contralateral gland without enlargement.^[20]^

### 2.3. Statistical analyses

Data distribution was assessed using the Shapiro–Wilk test and the variables whose measurement data conformed a normal distribution were analyzed by independent sample *t* test and reported as mean ± standard deviation (SD). Non normally distributed variables were analyzed by Mann–Whitney test and expressed as median (quartile range). Counts data was analyzed by chi square test and expressed in frequency (percentage). Taking the existence of the dominant side as the dependent variable and the variable with *P* < .05 in univariate analysis as the independent variable, the related factors associated with UPA were analyzed by multivariate logistic regression. In the adrenal CT scan results of unilateral typical nodules, the dependent variable was whether it represented UPA and whether the discriminatory results from the CT scan and AVS were consistent. The variable with a *P* < .1 in univariate analysis represented the independent variable. The factors associated with the concordance between UPA, CT and AVS were analyzed by multifactorial logistic regression. A receiver operating characteristic (ROC) curve was used for analysis and expressed as the area under the curve (AUC) and 95% confidence interval (95% CI). All the above analyses were analyzed using SPSS (IBM Corporation, Armonk, NY) and Medcalc (MedCalc 15.2.2 version, MedCalc Inc., Mariakerke, Belgium).

## 3. Result

### 3.1. Baseline data

We included 269 patients with PA, aged 46.24 ± 10.64, into the study, including 113 males (42.01%) and 156 females (57.99%), with a maximum systolic blood pressure of 184 (172–200); lowest serum potassium 3.38 (2.96–3.69) and 112 (41.64%) cases had typical nodular changes and 152 cases had UPA diagnosed by AVS. CT imaging results showed that 49, 177, and 43 cases were bilateral normal, unilateral abnormal and bilateral abnormal respectively.

### 3.2. Analysis of related factors of unilateral aldosteronism

In Table 1, the AVS results show the association between UPA and age (44.78 ± 10.49 vs 47.37 ± 10.63; *P* = .047), maximum systolic blood pressure [180 (163–190.5) vs 188 (180–200.75); *P* < .001], history of stroke [8 (6.84%) vs 33 (21.71%); *P* = .001], lowest serum potassium [3.47 (3.0–3.73) vs 3.33 (2.82–3.67); *P* = .03], and typical nodules [37 (31.62%) vs 75 (49.34%); *P* = .003] and these correlation showed statistically significant differences. The proportion of both male and female patients with UPA was higher than those with BPA and there were proportionately more female patients, overweight patients [(103 (67.76%) vs (77 (65.81%)] and family history of hypertension [85 (55.92%) vs 71 (60.68%)]. There was no significant difference in the maximum diastolic blood pressure between the two groups. In terms of renal function, the glomerular filtration and endogenous creatinine clearance rates in patients with BPA were slightly higher than those in patients with UPA. In terms of left ventricular mass index, UPA is relatively high. There was no significant difference in screening aldosterone and renin, while the ARR value of patients with UPA was higher than BPA. For the CT scan results, the proportion of bilateral abnormalities in UPA patients was higher [104 (68.42%) vs 73 (62.40%)].

**Table 1:** Univariate and multivariate analysis of patients with unilateral aldosteronism.

Variables	BPA (N = 117)	UPA (N = 152)	Univariate analysis	Multivariate analysis	
			*P* value	OR (95% CI)	*P* value
Sex, n (%)					
Male	49 (41.88)	64 (42.11)	.97		
Female	68 (58.12)	88 (57.89)			
Age (yr)	44.78 ± 10.49	47.37 ± 10.63	**.047**		
BMI ≥ 24, n (%)	77 (65.81)	103 (67.76)	.736		
MaxSBP (mm Hg)	180 [163–190.5]	188 [180–200.75]	**<.001**	1.03 (1.01–1.04)	**<.001**
MaxDBP (mm Hg)	110 [100–115]	110 [100–120]	.305		
Duration of hypertension (yr)	2 [0.5–7.5]	3 [1–8]	.092		
Family history of hypertension, n (%)	71 (60.68)	85 (55.92)	.433		
Dyslipidemia, n (%)	51 (43.59)	52 (34.21)	.117		
Hyperuricemia, n (%)	17 (14.53)	12 (7.89)	.082		
History of stoke, n (%)	8 (6.84)	33 (21.71)	**.001**	2.61 (1.11–6.16)	**.028**
Lowest Potassium (mEq/L)	3.47 [3.0–3.73]	3.33 [2.82–3.67]	**.03**		
GFR (mL/min)	95.62 [81.20–106.76]	94.66 [79.05–109.38]	.784		
Ccr (mL/min)	107.48 ± 28.43	101.69 ± 30.44	.113		
Aldosterone at screening (ng/dL)	21.73 [18.88–25.96]	21.25 [17.88–27.36]	.943		
PRA at screening (ng/mL/h)	0.67 (0.39–1.00)	0.75 (0.44–1.17)	.137		
ARR at screening [(ng/dL)/(ng/mL/h)]	40.6 [27.22–69.33]	43.06 [26.32–105.51]	.252		
LVMI (g/m^2^)	127.64 [109.9–149.51]	129.4 [115.89–148.12]	.368		
Typical nodules, n (%)	37 (31.62)	75 (49.34)	**.003**	1.90 (1.12–3.22)	**.017**
CT scanning findings, n (%)					
Bilaterally normal	25 (21.37)	24 (15.79)	.472		
Bilaterally abnormal	73 (62.40)	104 (68.42)			
Unilateral abnormality	19 (16.24)	24 (15.79)			

Typical nodules is Adrenal CT shows nodule ≥ 8 mm. The bold values represent statistically significant differences (*P* < .05).

ARR = plasma aldosterone renin ratio, BMI = body mass index, Ccr = creatinine clearance rate, GFR = glomerular filtration rate, LVMI = left ventricular mass index, MaxDBP = maximum diastolic blood pressure, MaxSBP = maximum systolic blood pressure, PRA = plasma renin activity.

**Figure 1.**
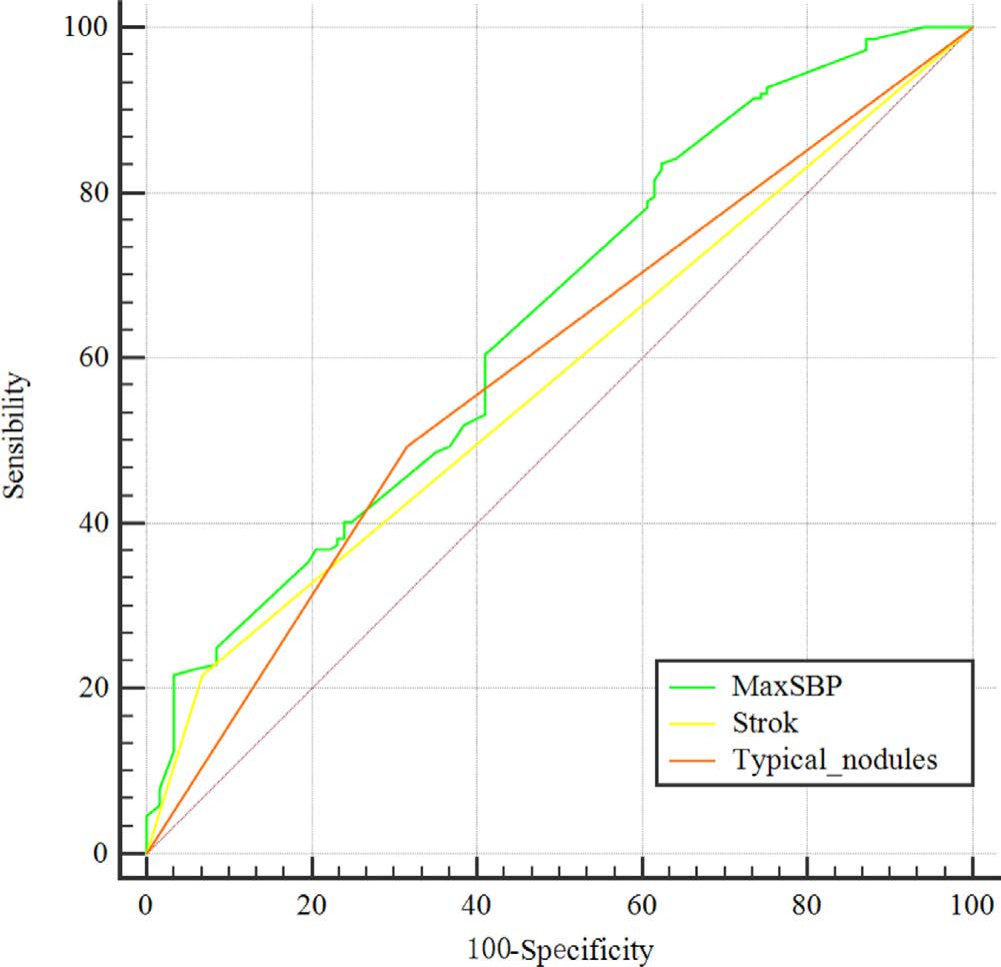
ROC comparison of maximum systolic blood pressure, stroke history and typical nodules; The AUC of the maximum systolic blood pressure was 0.647, which was the largest relative to stroke (AUC = 0.574) and typical nodules (AUC = 0.589). The dotted line represents the reference line. AUC = area under the curve, MaxSBP = maximum systolic blood pressure, ROC = receiver operating characteristic.

Multivariate logistic regression analysis showed that the maximum systolic blood pressure (OR = 1.03, *P* < .001), stroke history (OR = 2.61, *P* = .028) and the presence of typical nodules (OR = 1.90, *P* = .017) represented the independent related factors of UPA (Table 1). In addition, the comparative analysis for the ROC curves of related factors with *P* < .05 in multivariate logistic regression showed that (Fig. 1) the AUC for the maximum systolic blood pressure was 0.647 (95% CI: 0.582–0.713), for typical nodules it was 0.589 (95% CI: 0.530–0.647) and the AUC for stroke history was 0.574 (95% CI: 0.534–0.614).

### 3.3. Analysis of related factors in patients with UPA in the unilateral nodule group

**Figure 2.**
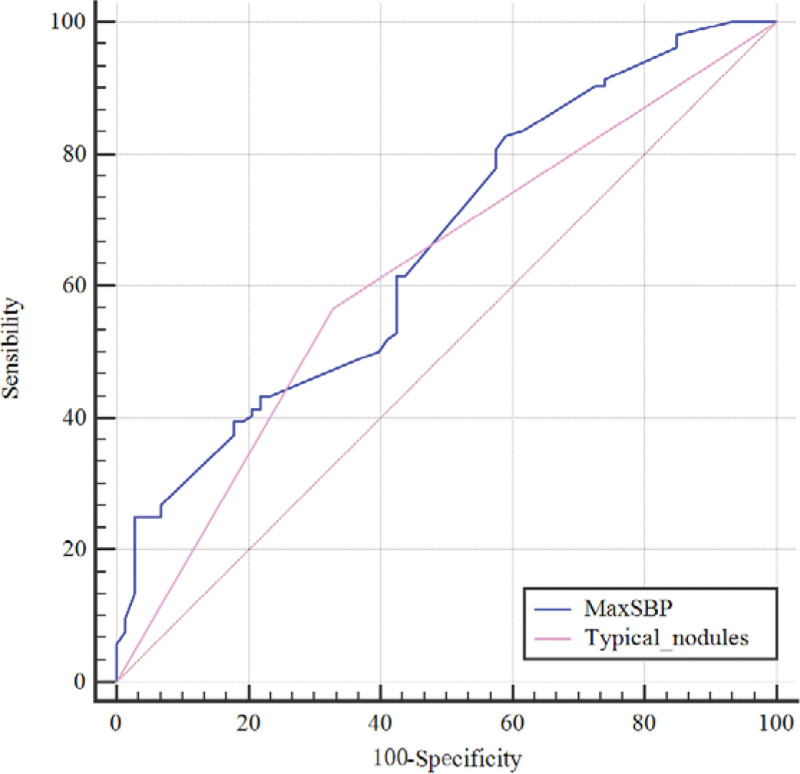
ROC comparison for the maximum systolic blood pressure and typical nodules; the AUC of the maximum systolic blood pressure was 0.66, which was larger than that of typical nodules (AUC = 0.619). The dotted line represents the reference line. AUC = area under the curve, MaxSBP = maximum systolic blood pressure, ROC = receiver operating characteristic.

Among the 177 patients in the unilateral nodule group, there were 73 and 104 patients with BPA and UPA, respectively. Compared to the BPA patients, there were significant differences in the maximum systolic blood pressure (*P* < .001), stroke history (*P* = .004) and typical nodules (*P* = .002) in UPA patients. Furthermore, multivariate logistic regression analysis showed that patients with a higher maximum systolic blood pressure (OR = 1.03, *P* < .001) and typical nodules (OR = 2.37, *P* = .008) were more likely to be diagnosed with UPA (Table 2). In addition, comparative analysis of the ROC curves for related factors with *P* < .05 in the multivariate logistic regression showed that (Fig. 2) the AUC of the maximum systolic blood pressure was 0.66 (95% CI: 0.579–0.74) and the AUC for typical nodules was 0.619 (95% CI: 0.547–0.692).

**Table 2:** Univariate and multivariate analyses of unilateral primary aldosteronism in the unilateral nodule group.

Variables	BPA (N = 73)	UPA (N = 104)	Univariate analysis	Multivariate analysis	
			*P* value	OR (95% CI)	*P* value
Sex, n (%)					
Female	41 (56.16)	60 (57.69)	.84		
Male	32 (43.84)	44 (42.31)			
Age (yr)	44.4 ± 10.54	47.16 ± 10.74	**.091**		
BMI ≥ 24, n (%)	47 (64.38)	69 (66.35)	.787		
MaxSBP (mm Hg)	180 (160–190)	188.5 (180–205.75)	**<.001**	1.03 (1.02–1.05)	**<.001**
MaxDBP (mm Hg)	110 (100–119.5)	110 (100–120)	.754		
Duration of HTN (yr)	2 (0.5–7.0)	3 (1–8)	**.067**		
Family history of hypertension, n (%)	45 (61.64)	56 (53.85)	.302		
Dyslipidemia, n (%)	31 (42.47)	36 (34.62)	.289		
Hyperuricemia, n (%)	10 (13.70)	7 (6.73)	.121		
History of stoke, n (%)	5 (6.85)	24 (23.08)	**.004**		
Lowest potassium (mEq/L)	3.4 (2.99–3.69)	3.29 (2.74–3.57)	**.05**		
GFR (mL/min)	94.33 (78.47–105.93)	98.87 (81.47–111.76)	.341		
Ccr (mL/min)	107.24 ± 28.53	102 ± 30.73	.252		
Aldosterone at screening (ng/dL)	21.9 (18.22–26.17)	21.02 (17.57–27.13)	.589		
PRA at screening (ng/mL/h)	0.71 (0.39–1.0)	0.82 (0.53–1.18)	**.095**		
ARR at screening [(ng/dL)/(ng/mL/h)]	36.76 (26.81–67.61)	41.26 (26.80–89.38)	.318		
LVMI (g/m^2^)	127.82 (113.17–145.09)	133.82 (118.39–151.33)	**.082**		
Typicalnodule, n (%)	24 (32.88)	59 (56.74)	**.002**	2.43 (1.26–4.66)	**.008**

Typical nodules are Adrenal CT showing nodules ≥ 8 mm. The bold values represent statistically significant differences (*P* < .05).

ARR = plasma aldosterone renin ratio, BMI = body mass index, BPA = bilateral primary aldosteronism, Ccr = creatinine clearance rate, GFR = glomerular filtration rate, LVMI = left ventricular mass index, MaxDBP = maximum diastolic blood pressure, MaxSBP = maximum systolic blood pressure, PRA = plasma renin activity, UPA = unilateral primary aldosteronism.

### 3.4. Analysis of related factors related to consistency between AVS and CT results in the unilateral nodule group

**Figure 3.**
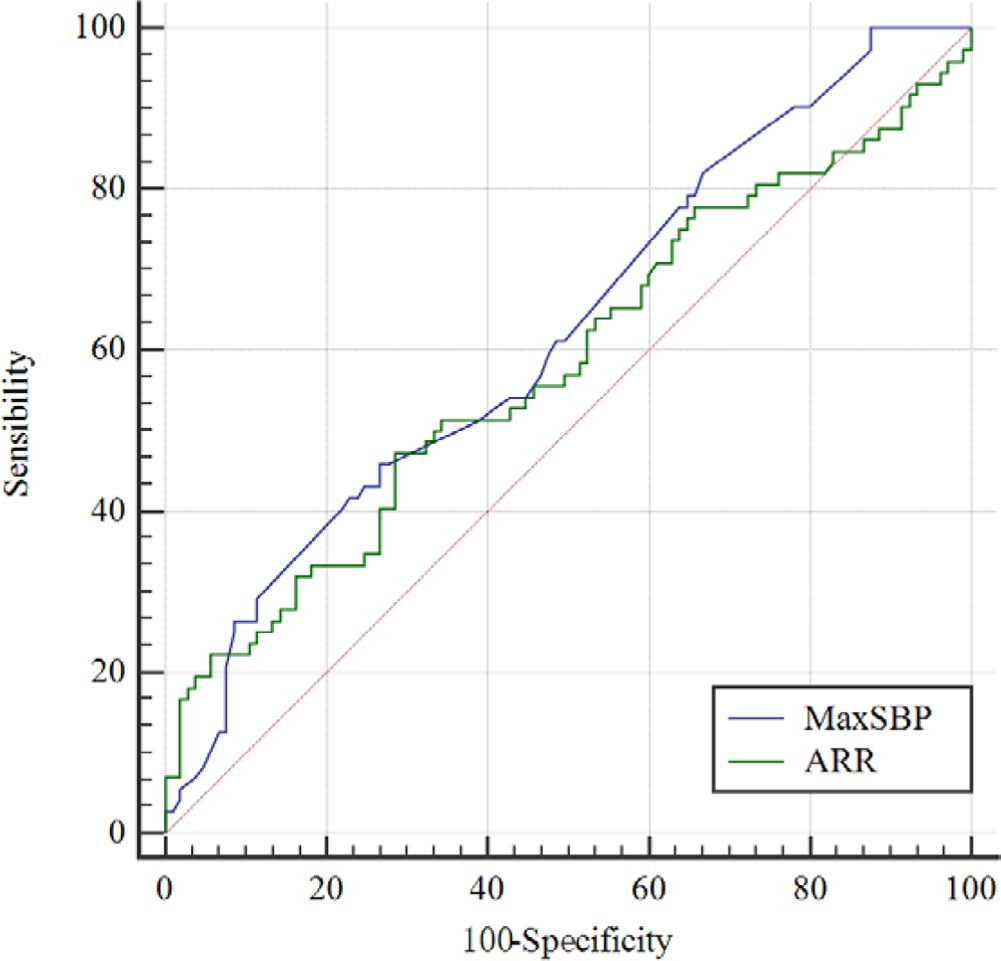
ROC comparison for maximum systolic blood pressure and ARR and the AUC for the maximum systolic blood pressure was 0.623, which was larger than that of ARR (AUC = 0.583). The dotted line is the reference line. ARR = ratio of aldosterone to renin, AUC = area under the curve, MaxSBP = maximum systolic blood pressure, ROC = receiver operating characteristic.

The nodal side and the occurrence of lateralization were determined to be consistent in 72 of the cases (40.68%, 72/177), and the nodal side contralateral to the occurrence of lateralization was determined to be inconsistent in 105 of the cases (59.32%, 105/177). There were significant differences in the maximum systolic blood pressure (*P* = .005), history of stroke (*P* = .01) and lowest potassium (*P* < .001) between the consistent group and the inconsistent group. In addition. There was a greater ARR (OR = 1.001, *P* = .027) in patients with unilateral nodules with a higher likelihood of concordance between CT and AVS findings (Table 3). In addition, the comparative analysis of the ROC curve for the correlation factors with *P* < .05 in the multivariate logistic regression showed that (Fig. 3) the AUC for the maximum systolic blood pressure was 0.623 (95% CI: 0.539–0.706) and the AUC of ARR was 0.583 (95% CI: 0.494–0.671).

**Table 3:** Univariate and multivariate analyses of consistent computed tomography and adrenal venous sampling results in patients with unilateral nodules.

Variables	AVS and CT findings are inconsistence (N = 105)	AVS and CT findings are consistence (N = 72)	Univariate analysis	Multivariate analysis	
			*P* value	OR (95% CI)	*P* value
Sex, n (%)					
Female	57 (54.29)	44 (61.11)	.367		
Male	48 (45.71)	28 (38.89)			
Age (yr)	45.83 ± 9.94	46.31 ± 11.83	.772		
BMI ≥ 24, n (%)	70 (66.67)	46 (63.89)	.702		
MaxSBP (mm Hg)	180 (170–195.5)	190 (180–209.25)	**.005**	1.04 (1.02–1.06)	**<.001**
MaxDBP (mm Hg)	110 (100–120)	110 (100–119.25)	.856		
Duration of HTN (yr)	2 (0.5–7.0)	3 (1–8)	.115		
Family history of hypertension, n (%)	58 (55.24)	43 (59.72)	.554		
Dyslipidemia, n (%)	49 (46.67)	27 (37.5)	.936		
Hyperuricemia, n (%)	12 (11.43)	5 (6.94)	.32		
History of stoke, n (%)	11 (10.48)	18 (25)	**.01**		
Lowest potassium (mEq/L)	3.41 (3.03–3.69)	3.03 (2.65–3.44)	**<.000**		
eGFR (mL/min)	95.08 (79.23–106.28)	100.44 (81.47–119.42)	.122		
Ccr (mL/min)	104.42 ± 28.60	103.80 ± 31.84	.890		
Aldosterone at screening (ng/dL)	21.44 (18.08–26.09)	22.03 (17.89–28.11)	.319		
PRA at screening (ng/mL/h)	0.75 (0.42–1.02)	0.83 (0.59–1.11)	.354		
ARR at screening [(ng/dL)/(ng/mL/h)]	36.76 (25.49–68.92)	48.88 (28.19–108.21)	**.062**	1.01 (1.001–1.01)	**.024**
LVMI (g/m^2^)	128.04 (113.97–147.41)	132.81 (118.52–150.94)	.284		

The bold values represent statistically significant differences (*P* < .05).

ARR = plasma aldosterone renin ratio, AVS = adrenal venous sampling, BMI = body mass index, Ccr = creatinine clearance rate, CT = computed tomography, GFR = glomerular filtration rate, LVMI = left ventricular mass index, MaxDBP = maximum diastolic blood pressure, MaxSBP = maximum systolic blood pressure, PRA = plasma renin activity.

## 4. Discussion

Primary aldosteronism is a clinical syndrome characterized by hypertension with or without hypokalemia. The purpose of subtype diagnosis is to distinguish unilateral lesions or bilateral lesions, to select a suitable treatment regimen.^[19]^ Therefore, determining whether lateralization occurs in patients with PA is crucial for this subtype diagnosis. In this study, the results of multiple logistic regression and AUC analyses of the data set showed that the maximum systolic blood pressure, history of stroke, and the presence of typical nodules were found to be relevant factors for UPA. After this, we explored further patient subgroups based on clinical CT imaging, and findings from the results showed differences in maximum systolic blood pressure between patients with UPA and BPA, suggesting that the higher the maximum systolic blood pressure, the greater the possibility of UPA.

Our findings suggest that UPA is closely associated with stroke and maximum systolic blood pressure, especially in moderate to severe hypertension. This may be due to sodium retention, and hypertension due to increased secretion of aldosterone. It has been shown that biochemical treatment of hypokalemia and normalization of aldosterone levels after adrenalectomy is almost certainly involved. However, not all patients are completely cured of hypertension after adrenalectomy.^[21]^ This suggests that blood pressure is a risk factor in its own right. To the best of our knowledge, this is the first report demonstrating maximum systolic blood pressure is an important factor in UPA. Takeda et al.^[22]^ also reported the incidence of vascular complications in a series of 136 patients with primary aldosteronism, with 31 (22–8%) cases developing cerebrovascular injury and myocardial infarction over a mean observation period of 5 to 7 years. Tomaschitz et al^[23]^ showed that higher plasma aldosterone concentrations (PAC) were strongly associated with increased stroke mortality, even in the absence of primary aldosteronism. However, their study was only focused on a specific population (patients who underwent coronary angiography). Takeda et al^[22]^ demonstrated that, in a prospective study of 883 cases, 45 cases of stroke occurred during the average follow-up of 10.9 years. The study found that high ARR, or relative aldosterone excess, was a predictor of stroke in conditions of high sodium intake. In recent years, Tang et al^[24]^ conducted a survey of the prevalence and clinical characteristics of PA in 116 young adults with acute stroke, and found that the prevalence of PA was at least 12.9% among adults with stroke under 45 years old. Among young patients with a history of hypertension and stroke, the prevalence of PA was 21.2%, which was far higher than the general population and stubborn hypertension according to previous reports. Compared to patients without PA, patients with PA have higher blood pressure levels and an increased incidence of hypertension and hypokalemia. This suggests the need for more rigorous screening for PA in adults with acute stroke episodes under 45 years of age, especially in those with severe hypertension and hypokalemia. Interestingly, relevant experiments and human studies have confirmed that excessive aldosterone can promote cerebrovascular oxidative stress, inflammation, and endothelial dysfunction, thereby increasing the risk of stroke, independent of blood pressure and other risk factors.^[25–27]^It has been reported that the clinical scores from Kupers et al^[28]^ on the diagnosis of proto aldehyde subtypes are related to typical adenomas, which is consistent with the results of our study. Previous studies however, have shown that AVS is an unnecessary investigation for patients with typical unilateral imaging manifestations in younger age cohorts^[29,30]^

Patients with unilateral nodal PA requiring surgical resection represent the most challenging clinical decisions. In our study, multivariate logistic regression analysis in the unilateral nodule group showed that patients with PA and a higher maximum systolic blood pressure and typical nodules were more likely to be diagnosed as UPA. Moreover, multivariate logistic regression analysis showed that there was significant difference in the highest systolic blood pressure and ARR between CT and AVS. It has been reported that the score regarding the prediction for the use of procedural subtypes consisted of serum potassium, plasma aldosterone, and ARR.^[31]^ Unfortunately, ARR was not an independent correlate when discriminating for UPA.

## 5. Limitation

Our study also has some limitations: firstly, similar to almost all other studies on PA, retrospective designs may limit the universality of our results, and further prospective studies are needed to confirm our findings. Secondly, the research object comes from a single center research in Guangxi, China, and large sample data are needed to further verify the research results in the future.

## 6. Conclusion

In conclusion, the maximum systolic blood pressure, stroke history and typical nodules are important related factors for the diagnosis of different PA subtype. When diagnosing of subtypes of patients with PA, a combination of medical history and imaging data is recommended. At the same time, patients with PA with no obvious CT lesions or bilateral lesions are recommended to be divided into different subgroups for clinical decision-making according to the imaging results. Centers that are eligible for AVS should perform it to clarify the functional staging and diagnosis of PA where possible.

## Acknowledgments

The authors thank the participants and participating doctors from the First Affiliated Hospital of Guangxi Medical University. Also, the authors would like to express their gratitude to EditSprings (https://www.editsprings.cn/) for the expert linguistic services provided.

## Author contributions

YM, KC, AX, and YL are responsible for data collection. YM conducted data sorting, statistical analysis and manuscript composition. JH contributed to the study conception and design. All authors read and approved the final version of the manuscript.
